# Evaluation of the metastatic status of lymph nodes identified using axillary reverse mapping in breast cancer patients

**DOI:** 10.1186/1477-7819-10-233

**Published:** 2012-11-01

**Authors:** Katsumi Ikeda, Yoshinari Ogawa, Hisateru Komatsu, Yoshihiro Mori, Akira Ishikawa, Takayoshi Nakajima, Gou Oohira, Shinya Tokunaga, Hiroko Fukushima, Takeshi Inoue

**Affiliations:** 1 Department of Breast Surgical Oncology, Osaka City General Hospital, 2-13-22 Miyakojima-hondori, Miyakojima-ku, Osaka, 534-0021, Japan; 2 Department of Gastroenterological Surgery, Osaka City General Hospital, Osaka, Japan; 3 Department of Clinical Oncology, Osaka City General Hospital, Osaka, Japan; 4 Department of Pathology, Osaka City General Hospital, Osaka, Japan

**Keywords:** Breast cancer, Axillary reverse mapping, Fine needle aspiration cytology, Fluorescence image

## Abstract

**Background:**

Axillary reverse mapping (ARM) is a new technique to preserve upper extremity lymphatic pathways during axillary lymph node dissection (ALND), thereby preventing lymphedema patients with breast cancer. However, the oncologic safety of sparing the nodes identified by ARM (ARM nodes), some of which are positive, has not been verified. We evaluated the metastatic status of ARM nodes and the efficacy of fine needle aspiration cytology (FNAC) in assessing ARM node metastasis.

**Methods:**

Sixty patients with breast cancer who underwent ARM during ALND between January 2010 and July 2012 were included in this study. Twenty-five patients were clinically node-positive and underwent ALND without sentinel lymph node biopsy (SLNB). Thirty-five patients were clinically node-negative but sentinel node-positive on the SLND. The lymphatic pathway was visualized using fluorescence imaging with indocyanine green. ARM nodes in ALND field, whose status was diagnosed using FNAC, were removed and processed for histology. We evaluated the correlation between the cytological findings of FNAC and the histological analysis of excised ARM nodes.

**Results:**

The mean number of ARM nodes identified per patient was 1.6 ±0.9 in both groups. In most patients without (88%) and with (79%) SLNB, the ARM nodes were located between the axillary vein and the second intercostobrachial nerve. FNAC was performed for 45 ARM nodes, 10 of which could not be diagnosed. Six of the patients without SLNB (24%) and onewith SLNB (3%) had positive ARM nodes. Of these sevenpatients, four had >3 positive ARM nodes. There was no discordance between the cytological and histological diagnosis of ARM nodes status.

**Conclusions:**

Positive ARM nodes were observed in the patients not only with extensive nodal metastasis but also in those with a few positive nodes. FNAC for ARM nodes was helpful in assessing ARM nodes metastasis, which can be beneficial in sparing nodes essential for lymphatic drainage, thereby potentially reducing the incidence of lymphedema. However, the success of sampling rates needs to be improved.

## Background

Sentinel lymph node biopsy (SLNB) is currently considered as a standard of care to determine the spread of cancer in patients with early stage breast cancer who have clinical node-negative axilla. Recently, the American College of Surgeons Oncology Group’s (ACOSOG) Z0011 randomized trial reported that axillary lymph node dissection (ALND) in patients with clinically node-negative axilla could be omitted despite the presence of a few positive sentinel nodes [1]. However, recent meta-analysis of local treatments for breast cancer without ACOSOG Z0011 trial showed that achieving good local control with appropriate adjuvant therapy could improve the prognosis of the patients with breast cancer [2]. Although the significance of performing ALND for patients with clinically node-negative and sentinel node-positive must be debated, ALND has been still the standard treatment for patients with clinically node-positive axilla. The morbidities associated with ALND are of concern with regard to the benefit for the patient. Upper extremity lymphedema is one of the most severe complications in patients with breast cancer who undergone ALND, and this complication has been reported in 6% to 49% of patients [3-6]. Breast cancer survivors with lymphedema may experience different degrees of physical and emotional disability that can severely affect the quality of life [7]. Recently, the axillary reverse mapping (ARM) has been developed to preserve arm lymphatic drainage during ALND or SLNB [8-11]. Boneiti *et al*. [12] reported that lymphedema developed in two of 12 patients following the lymph node and/or lymphatic duct removal in lymph drainage pathway identified by ARM. By comparison, lymphedema did occur in patients whose ARM nodes remained intact during ALND. Thus, sparing ARM nodes may reduce the incidence of lymphedema after ALND [8-14]. The ARM procedure is based on the hypothesis that the lymphatic pathway in the upper extremities would not involve metastasis from the primary breast cancer nest [15]. However, previous studies reported metastatic involvement in the ARM nodes in up to 43% of patients [8-14]. Thus, the oncologic safety of retaining lymph nodes or lymphatic ducts identified by ARM is an important issue for an advancement of ARM as a standard of care. To our knowledge, there are currently no reports on any method to assess the oncologic safety of preserving ARM nodes during ALND. Fine needle aspiration cytology (FNAC) for axillary nodes is a popular and variable technique to diagnosis lymph nodes metastasis before surgery [16]. We analyzed nodal status of ARM nodes and evaluated the efficacy of intraoperative FNAC for ARM nodes to appraise the oncologic safety of ARM node sparing during ALND.

## Methods

### Patients

Between January 2010 and July 2012, 372 patients with breast cancer underwent breast surgery at our hospital. In total, 116 of 372 patients required ALND, and 80 of these patients underwent ARM and were enrolled in the present study. Twenty patients who received primary systemic treatment (chemotherapy or endocrine therapy) were excluded. In total, 60 patients were included in the final evaluation. The study was approved by the institutional review board in our hospital. Written informed consent was obtained from all patients that participated in the study. We included patients with clinically or cytologically node-positive axilla determined by FNAC and patients with clinically node-negative axilla who had positive sentinel nodes revealed by SLNB. Any patients with an iodine allergy were ineligible for inclusion because of the use of indocyanine green as a tracing agent (ICG; Diagnogreen; Daiichi Sankyo, Co., Ltd., Tokyo, Japan).

The mean ages of the patients without SLNB (with clinically node-positive axilla) and with SLNB (with clinically node-negative axilla and positive sentinel nodes) were 57 ± 11 years and 62 ± 12 years, respectively. The mean body mass indexes of the patients in two groups were 23.2 ± 4.2 (without SLNB) and 23.5 ± 5.4 (with SLNB). Characteristics of patients in the two groups are shown in Table 1. Patients with SLNB had clinical stage I (46%) and IIA (51%) breast cancer, whereas the clinical stages of the patients without SLNB were IIA (32%), IIB (44%), IIIA (8%), IIIB (4%), and IIIC (12%). Clinical breast cancer stage was determined according to the Union for International Cancer Control (UICC) TNM classification and staging [17].

**Table 1:** A comparison of clinicopathological features between without SLNB and with SLNB groups

	**Patients without SLNB (*n*=25)**	**Patients with SLNB (*n*=35)**	***P *value**
Age (years)	57.2±11.3	61.5±12.1	*NS*
BMI	23.2±4.2	23.5±5.4	*NS*
T			0.09
0	2 (8)	0 (0)	
1	6 (24)	17 (48.5)	
2	14 (56)	17 (48.5)	
3	2 (8)	0 (0)	
4	1 (4)	1 (3)	
N			<0.001
0	0	35 (100)	
1	20 (80)	0 (0)	
2	2 (8)	0 (0)	
3	3 (12)	0 (0)	
Clinical stage			<0.001
I	0	16 (46)	
IIA	8 (32)	18 (51)	
IIB	11 (44)	0	
IIIA	2 (8)	0	
IIIB	1 (4)	1 (3)	
IIIC	3 (12)	0	
Histology			0.33
IDC	23 (92)	35 (100)	
Others	2^a^ (8)	0 (0)	
ER			0.10
Negative	7 (28)	3 (9)	
Positive	18 (72)	32 (91)	
HER-2			0.24
Negative	15 (60)	26 (74)	
Positive	10 (30)	9 (26)	

Numbers in brackets are percentages.

^a^The histology of two patients’ breast cancer was invasive micropapillary carcinoma and apocrine carcinoma.

ALND, Axillary lymph node dissection; BMI, Body mass index; ER, Estrogen receptor; HER-2, Human epidermal growth factor receptor 2; IDC, Invasive ductal carcinoma; NS, Not significant; SLNB, Sentinal node biopsy.

The patients without SLNB had significantly more advanced breast cancer than patients with SLNB (*P* <0.001). Estrogen receptor status and human epidermal growth factor receptor type 2 status were almost similar between the two groups.

### Sentinel node biopsy procedure

In the patients with clinically node-negative axilla, 1 to 2 mL of indigocarmine (Daiichi Sankyo, Co., Ltd., Tokyo, Japan) was administered via intra-dermal injection to the areolar region with a 26-gauge needle after the induction of general anesthesia and before the patient was prepared for surgery. ALND was performed if either frozen sections or touch imprint cytology of the sentinel nodes revealed metastasis. The procedure for ALND is described below.

### Axillary reverse mapping and intraoperative fine needle aspiration cytology for ARM nodes

Patients with clinically node-positive axilla initially received an intra-dermal injection of 0.5 to 1.0 mL (5 mg/mL) ICG at the upper and inner ipsilateral arm using a 1-mL syringe with a 26-gauge needle under general anesthesia before preparation for breast surgery. After massaging for approximately 5 min, the fluorescence signal was detected using an invisible near-infrared fluorescence imaging system (PDE™; Photo Dynamic Eye; Hamamatsu Photonics Company, Hamamatsu, Japan) as flowing to the axilla from the upper extremity. Patients who had positive sentinel nodes as shown on SLNB received an ICG injection before ALND in a manner similar to that for patients with clinically node-positive axilla.

**Figure 1:**
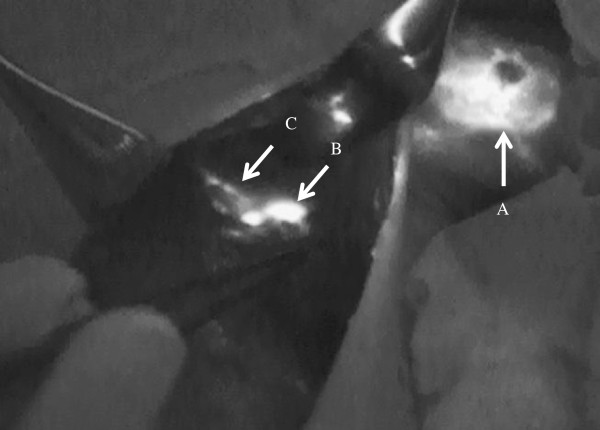
**Fluorescence imaging using the Photo Dynamic Eye System.** (**A**, **B**, **C**) White arrows indicate the site injected with indocyanine green, ARM lymph nodes, and ARM lymphatic ducts, respectively.

**Figure 2:**
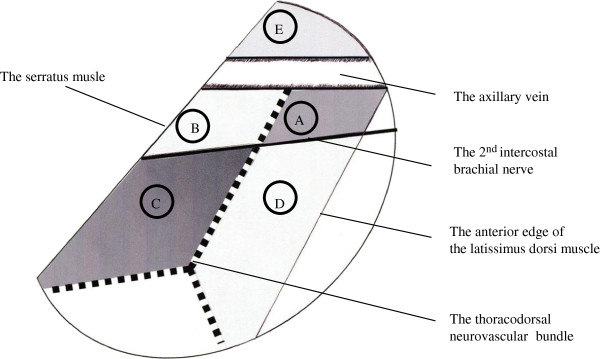
**Mapping of the ARM lymph nodes in the axilla.** Field A is the area between the axillary vein and the second intercostobrachial nerve and between the anterior edge of the latissimus dorsi muscle and the thoracodorsal neurovascular bundle. Field B is the area medially adjacent to field A and close to the anterior serratus muscle (the chest wall). Field C is the area surrounding the second intercostobrachial nerve, the anterior serratus muscle, and the thoracodorsal neurovascular bundle. Field D is the area below field A and laterally adjacent to field C. Field E is the area above the axillary vein.

**Figure 3:**
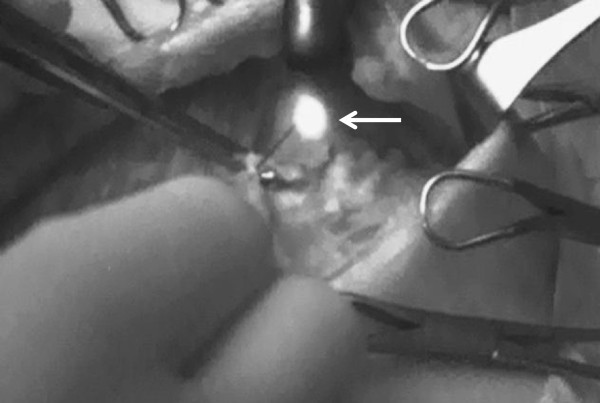
**The picture of performing intraoperative FNAC for ARM node.** This picture shows performing intraoperative FNAC for an ARM node (indicated with a white arrow) with a 23-gauge needle in the surgical field.

After performing lumpectomy or mastectomy for breast cancer, we started to explore ARM nodes in the axillary field. ARM nodes and lymphatic ducts were identified as fluorescent objects in the axilla even if they did not exhibit a green-colored stain (Figure 1). The locations of all identified ARM nodes were marked on the map according to the surgical landmarks of the axilla, which include the axillary vein, the thoracodorsal neurovascular bundle, and the second intercostobrachial nerve (Figure 2). The limits of ALND were defined by the axillary vein, superiorly; the anterior serratus muscle, medially; and the anterior edge of the latissimus dorsi muscle, laterally. When fluorescent ARM nodes were located in the ALND field, they were separately removed after performing FNAC to evaluate the pathological findings. FNAC was performed using a 23-gauge needle attached to a 10-mL aspirating syringe (Figure 3). On the other hand, when identified ARM nodes were outside the ALND field, they were spared, and we did not perform FNAC.

### Pathology evaluation

We retrospectively evaluated the correlation between the cytological findings on FNAC and the histological results of removed ARM nodes. Cytological findings of classic Papanicolau and Giemsa staining were interpreted by two pathologists with an established experience in breast cytology. The cytological results were reported as negative, suspicious, positive for malignancy, or inadequate for diagnosis. Histological findings were diagnosed using hematoxylin and eosin staining at the maximum longitudinal section of each node.

### Statistical analysis

Stat Mate version IV for Windows was used for data analyses. To estimate the differences in the clinicopathological and categorical data associated with the ARM procedure between the groups with or without SLNB, we used the chi-square or the Mann-Whitney *U* test. Two-tailed *P* values <0.05 were considered statistically significant.

## Results

Of 60 patients, 25 underwent ALND because of clinically node-positive axilla (without SLNB group), whereas 35 required ALND because of positive sentinel nodes on SLNB (with SLNB group). Fluorescence imaging using the PDE™ System identified ARM nodes and lymphatic ducts in 22 (88%) and 26 (74%) patients among the patients without SLNB and among those with SLNB, respectively. The total number of identified ARM nodes were 35 in the without SLNB group and 42 in the with SLNB group. The mean number of identified ARM nodes per person was 1.6± 0.9 in both groups.

In most patients, the ARM nodes were located between the axillary vein and the second intercostobrachial nerve (Figure 2). Five ALND fields were described as follows: field A, the area between the axillary vein and the second intercostobrachial nerve, and close to the anterior edge of the latissimus dorsi muscle; field B, the area medially adjacent to field A and close to the anterior serratus muscle; field C, the area below the second intercostobrachial nerve and close to the anterior serratus muscle; field D, the area below the second intercostobrachial nerve and close to the anterior edge of the latissimus dorsi muscle; field E, the area above the axillary vein. Field distribution of ARM node for the two groups is shown in Table 2. The distribution of ARM nodes identified in patients without SLNB was as follows: field A, 63%; field B, 25%; field C, 6%; field D, 0%; field E, 6%. In comparison, the distribution of ARM nodes identified in patients with SLNB was as follows: field A, 60%; field B, 19%; field C, 2%; field D, 2%; field E, 17%. Four (16%) patients without SLNB and 11 (31%) patients with SLNB displayed nodes outside the ALND field that were not assessed by FNAC.

**Table 2:** Results of ARM in patients without SLNB and with SLNB

	**Patients without SLNB (*n*=25)**	**Patients with SLNB (*n*=35)**	***P *value**
Identification			
ARM node	22 (88)	26 (74)	0.33
ARM lymphatic duct	19 (76)	30 (86)	0.54
Preservation			
ARM node	4 (16)	11 (31)	0.29
ARM lymphatic duct	8 (32)	17 (49)	0.20
ARM node			
Identified number			*NS*
Total	35	42	
AVR±SD	1.6±0.85	1.6±0.9	
Location			0.46
A	22 (63)	25 (60)	
B	9 (25)	8 (19)	
C	2 (6)	1 (2)	
D	0 (0)	1 (2)	
E	2 (6)	7 (17)	
Metastasis			0.035
	6 (24)	1 (3)	
FNAC			
Tested node number	23 (66)	22 (52)	0.23
Negative	13 (57)	17 (77)	0.26
Positive	4 (17)	1 (5)	
Inadequate	6 (26)	4 (18)	

Numbers in brackets are percentages.

FNAC, Fine needle aspiration cytology; NS, Not significant; SLNB, Sentinel node biopsy.

Six (24%) patients without SLNB had positive ARM nodes, whereas only one (3%) patient with SLNB showed metastasis to the ARM node. The patients without SLNB had a significantly higher frequency of positive ARM nodes than those with SLNB (*P* = 0.035) (Table 2). The features of the seven patients with positive ARM nodes are summarized in Table 3. In all these cases, the ARM nodes were located in field A (Figure 2) and the metastatic characteristic of the ARM nodes was defined as macrometastasis (with foci >2 mm). Case 2 listed in Table 2 had a single positive ARM node located in field A (Figure 2), whereas no metastasis were noted in the other axillary nodes that were removed. In the patients without and with SLNB, FNAC was performed for 23 and 22 ARM nodes, respectively, of which 17 (74%) and 18 (82%) nodes could be diagnosed using were FNAC, respectively. The remainder of nodes could not be assessed because of inadequate material (Table 2). For the 35 ARM nodes that could be assessed by FNAC, no discordance was noted between the cytological assessments by FNAC and the histological results. There was no evidence of metastasis in the 30 ARM nodes diagnosed as a negative for malignancy by FNAC; on the other hand, five ARM nodes diagnosed as either suspicious or positive by FNAC involved breast cancer metastasis.

**Table 3:** Review of the cases with positive ARM nodes

**Case**	**Age (years)**	**Clinical stage (TNM)**	**Surgery**	**SLNB**	**Number / location**^**a**^**of ARM nodes**	**Results of FNAC**	**Positive / removed ARM nodes (*n*)**	**Positive / removed nodes in axilla (*n*)**	**Metastatic area in the ARM nodes**
1	40	IIB (T2N1M0)	Bp	-	1/ A	Positive	1/1	2/14	Mac
2	65	IIB (T2N1M0)	Bt	-	1/ A	Inadequate	1/1	1/13^b^	Mac
3	55	IIB (T2N0M0)	Bt	+	2/ A, E	Positive	1/1	9/18	Mac
4	70	IIIB (T4N1M0)	Bt	-	2/ A,B	Positive/Negative	1/2^c^	7/16	Mac
5	66	IIB (T2N1M0)	Bp	-	1/ A	Suspected	1/1	15/19	Mac
6	79	IIIC (T2N3M0)	Bt	-	1/ A	Suspected	2/2^d^	20/27	Mac
7	52	IIA (T0N1M0)	Ax	-	1/ A	Inadequate	1/1	2/17	Mac

^a^Location of ARM nodes; Field A, B, C, D, and E are presented in Figure 2.

^b^A single ARM node had metastasis of breast cancer although the other removed axillary lymph nodes had no metastasis.

^c^The metastatic ARM node (located in field A) was positive by FNAC, whereas another ARM node (located in field B) diagnosed as negative by FNAC did not involve metastatic foci pathologically.

^d^The removed ARM node included two lymph nodes microscopically and there were metastasis in both ARM nodes.

Bp, Lumpectomy; Bt, mastectomy; FNAC, Fine needle aspiration cytology; Mac, Macrometastasis; SLNB sentinel lymph node biopsy.

## Discussion

According to previous ARM studies using dyes and radioisotopes, the incidence of ARM nodes detect in patient populations were reported to be 50% to 91% [8-14]. The detection of ARM lymphatic ducts is more important than that of ARM nodes because the aim of ARM is to spare lymphatic pathways, which is not the case for the SLNB procedure. Noguchi *et al*. reported that the identification rate of ARM nodes and/or lymphatics was 88% using fluorescence navigation with ICG, and concluded that the fluorescence imaging technique was useful for detecting not only the ARM lymph nodes, but also the lymphatic ducts [13]. In the present study, the identification rates of ARM nodes and lymphatic ducts were 88% and 76% for patients without SLNB and 74% and 86% for patients with SLNB, respectively, which was similar to the results in previous studies. The fundamental concept of ARM procedure is based on the assumption that the lymphatic pathway from the upper extremity would not involve metastasis from the primary breast cancer [8,11,12]. However, the metastatic rates to ARM nodes were reported to be around 14% to 43% in recent feasibility studies [10,13,14]. The oncologic safety of preserving ARM nodes or lymphatic ducts is the most important issue for performing ARM in practice. Nos *et al*. [10] explained the assumptions concerning ARM node metastasis. The first assumption was that ARM nodes would belong to the central nodal group, which is highly related to the lymphatic pathway originating from the breast. If this was the case, the ARM procedure would have limited surgical benefit.

A further assumption was that the natural progression of breast cancer would lead a metastasis to ARM node following the other extensive nodal metastasis. In the present study, the patients with clinically node-positive axilla had a higher risk (24%) of having positive ARM nodes than those with clinically node-negative axilla (3%). Of 35 patients with SLNB, only one patient had a positive ARM node (Table 3). She had a silicone implant in the bilateral breast and it was difficult to estimate the nodal status using ultrasonography (US) and computed tomography before surgery. She had severe nodal metastasis (nine positive nodes) at levels I to III of the axilla.

In the seven patients with positive ARM nodes, four (57%) had extensive nodal metastasis (> 4 metastatic nodes). This result may support the notion that patients with breast cancer and extensive nodal metastasis would have positive ARM nodes following tumor progression. However, the other three patients also had a few positive nodes in the axilla. In these patients, there must be another reason why ARM node status was positive. In previous ARM studies, ARM nodes and/or ducts were observed in the sentinel biopsy field in 38% to 75% of all cases [11-13] and concordance between sentinel nodes and ARM nodes was reported in approximately 20% of the patients [13]. The positive ARM node in noted in case 2 (Table 3) was not located in the sentinel lymph node biopsy field, which seems to equate to field B or C (Figure 2), but rather to field A, where ARM nodes were often observed (Figure 2). This case may suggest that some ARM nodes belong to the breast central nodes and that metastasis to ARM nodes could occur in the patients with a few positive axillary nodes. Therefore, it is important to confirm the metastatic status of ARM nodes in order to for those nodes to be retained during operation. Han *et al.* reported the use of FNAC or frozen biopsy of partial resection of suspicious ARM nodes to confirm oncologic safety if the surgeon suspected that those nodes were metastatic. Unfortunately, the detail information from that study concerning the FNAC results for ARM nodes is not available [18].

In practice, FNAC of axillary lymph nodes with US guidance is widely accepted as a useful procedure for providing preoperative information on nodal status [16]. A recent systematic review of the US-guided FNAC shows a sensitivity that varies between 30.6% and 62.9% and a specificity of 100% [19]. However, inadequate sampling is a potential limitation of FNAC for diagnosis. Ciatto *et al.*[16] reported that a sampling error might be corrected by repeated sampling and showed that sampling was insufficient in 10.8% of cases, which is comparable or lower than the inadequacy rate reported in other studies. In our study, the sampling error was 26% in the without SLNB group and 18% in with SLNB group. Micrometastasis in a lymph node was one of the causes for false-negative result in a previous study of FNAC for axillary lymph nodes [20]. However, the chief cause of our sampling error may be technical in nature because all patients had macrometastasis in the ARM nodes in the present study. Our sampling error rate was higher than that of FNAC by US-guided aspiration. Compared with an US-guided procedure, it may be difficult to obtain sufficient materials from lymph nodes in a surgical field using our method, because US imaging can be helpful in confirming the fine needle tract to the target. Repeat aspiration for ARM nodes may improve the success rate of sampling if sufficient material for diagnosis is not harvested after the first aspiration. The limitation of our study included the small number of enrolled patients and relative inexperience with the new procedure. Studies including a larger series of patients are required to determine the efficacy of FNAC for the preservation of ARM nodes during ALND.

## Conclusion

Metastasis to ARM nodes can occur both in patients with extensive nodal metastasis and in those with a few positive nodes. However, patients with clinically node-positive breast cancer had a significantly greater incidence of positive ARM nodes than those with clinically node-negative and sentinel node-positive breast cancer. FNAC for ARM nodes might be helpful for the assessment of metastasis in ARM nodes, but an improvement in the sample success rate is needed for a robust confirmation.

## Abbreviations

ACOSOG: The American College of Surgeons Oncology Group; ALND: Axillary lymph node dissection; ARM: Axillary reverse mapping; ARM nodes: The lymph nodes identified by the ARM procedure; FNAC: Fine needle aspiration cytology; ICG: Indocyanine green; SLNB: Sentinel lymph node biopsy; US: Ultrasonography.

## Competing interests

The authors declare that they have no competing interests.

## Authors’ contributions

KI participated in the study concept and design, and carried out data acquisition, analysis, and interpretation of data and drafting of the manuscript; YO participated in the study design and helped to draft the manuscript; HK, AI, TN, and GO participated in the study; ST carried out primary systemic treatment for the patients and participated in the study; HF and TI carried out the pathological examinations and interpretation of the specimens. All authors read and approved the final manuscript.

## References

[B1] Giuliano AE Hunt KK Ballman KV Beitsch P Whitworth PW Blumencranz PW Leitch AM Saha S McCall LM Morrow M Axillary dissection vs. no axillary dissection in women with invasive breast cancer and sentinel node metastasis: a randomized clinical trial JAMA 2011 305 569 575 10.1001/jama.2011.90 21304082 PMC5389857

[B2] Clarke M Collins R Darby S Elphinstone P Evans E Godwin J Gray R Hicks C James S MacKinnon E McGale P McHugh T Peto R Taylor C Wang Y Early Breast Cancer Trialists’ Collaborative Group (EBCTCG) Effects of radiotherapy and of differences in the extent of surgery for early breast cancer on local recurrence and 15-year survival: an overview of the randomized trials Lancet 2005 366 2087 2106 16360786 10.1016/S0140-6736(05)67887-7

[B3] Petrek JA Haalan MC Incidence of breast carcinoma-related lymphedema Cancer 1988 83 2776 2781 9874397 10.1002/(sici)1097-0142(19981215)83:12b+<2776::aid-cncr25>3.0.co;2-v

[B4] Petrek JA Senie RT Peters M Rosen PP Lymphedema in a cohort of breast carcinoma survivors 20 years after diagnosis Cancer 2001 92 1368 1377 10.1002/1097-0142(20010915)92:6<1368::AID-CNCR1459>3.0.CO;2-9 11745212

[B5] Nesvold IL Reinersten KV Fossa SD Dahl AA The relation between arm/shoulder problem and quality of life in breast cancer survivors: a cross-sectional and longitudinal study J Cancer Surviv 2011 5 62 72 10.1007/s11764-010-0156-4 20972640 PMC3040353

[B6] Nishio M Ogawa Y Ikeda K Okutani Y Nagahara H Sakurai K Tokunaga S Hamakawa T Nishiguchi Y Lymphedema following axillary lymph node dissection for breast cancer - about its etiology and risk factors Jpn J Breast Cancer 2007 22 469 474

[B7] Michael S Charikleia S Konstantinos K Lymphedema and breast cancer: a review of the literature Breast Cancer 2011 18 174 180 10.1007/s12282-010-0246-1 21331463

[B8] Thompson M Korourian S Henry-Tillman R Adkins L Mumford S Westbrook KC Klimberg VS Axillary reverse mapping (ARM): A new concept to identify and enhance lymphatic preservation Ann Surg Oncol 2007 14 1890 1895 10.1245/s10434-007-9412-x 17479341

[B9] Nos C Leiseur B Clough KB Lecuru F Blue dye injection in the arm in order to conserve the lymphatic drainage of the arm in breast cancer patients requiring an axillary dissection Ann Surg Oncol 2007 14 2490 2496 10.1245/s10434-007-9450-4 17549570

[B10] Nos C Kaufmenn G Clough KB Collignon MA Zerbib E Cusumano P Lecuru F Combined axillary reverse mapping (ARM) technique for breast cancer patients requiring axillary dissection Ann Surg Oncol 2008 15 2550 2555 10.1245/s10434-008-0030-z 18618185

[B11] Casabona F Bogliolo S Menada MV Sala P Villa G Ferrero S Feasibility of axillary reverse mapping during sentinel lymph node biopsy in breast cancer Ann Surg Oncol 2009 16 2459 2463 10.1245/s10434-009-0554-x 19506954

[B12] Boneti C Korourian S Bland K Cox K Adkins LL Henry-Tillman R Klimberg VS Axillary reverse mapping: mapping and preserving arm Lymphatics may be important in preserving lymphedema during sentinel lymph node biopsy J Am Coll Surg 2008 206 1038 1042 discussion 1042-1044 10.1016/j.jamcollsurg.2007.12.022 18471751

[B13] Noguchi M Yokoi M Nakano Y Axillary reverse mapping with indocyanine fluorescence imaging in patients with breast cancer J Surg Oncol 2010 101 217 221 20063370 10.1002/jso.21473

[B14] Bedrosian I Babiera GV Mittendorf EA Kuerer HM Pantoja L Hunt KK Krishnamurthy S Meric-Bernstam F A phase I study to assess the feasibility and oncologic safety of axillary reverse mapping in breast cancer patients Cancer 2010 116 2543 2548 20336790 10.1002/cncr.25096 PMC4406355

[B15] Noguchi M Axillary reverse mapping for breast cancer Breast Cancer Res Treat 2010 119 529 535 10.1007/s10549-009-0578-8 19842033

[B16] Ciatto S Brancato B Risso G Ambrogetti D Bulgaresi P Maddau C Turco P Houssami N Accuracy of fine needle aspiration cytology (FNAC) of axillary lymph node as a triage test in breast staging Breast Cancer Res Treat 2007 103 85 91 10.1007/s10549-006-9355-0 17033920

[B17] Sobin L Gospodarowicz M Witterkind C TNM Classification of Malignant Tumors 2009 7 Hoboken, NJ: Wiley 181 191

[B18] Han JW Seo YJ Choi JE Kang SH Lee SJ The efficacy of arm node preserving surgery using axillary reverse mapping for preventing lymphedema in patients with breast cancer J Breast Cancer 2012 15 91 97 10.4048/jbc.2012.15.1.91 22493634 PMC3318181

[B19] Alvarez S Anorbe E Alcorta P Lopez F Alonso I Cortes J Role of sonography in the diagnosis of axillary lymph node metastases in breast cancer: A systematic review Am J Roentgenol 2006 186 1342 1348 10.2214/AJR.05.0936 16632729

[B20] Krishnamurthy S Sneige N Bedi DG Edieken BS Fornage BD Kuerer HM Singletary SE Hunt KK Role of ultrasound-guided fine needle aspiration of indeterminate and suspicious axillary lymph nodes in the initial staging of breast carcinoma Cancer 2002 95 982 988 10.1002/cncr.10786 12209680

